# Perceived work-related stress and its associated factors among public secondary school teachers in Gondar city: a cross-sectional study from Ethiopia

**DOI:** 10.1186/s13104-020-4901-0

**Published:** 2020-01-17

**Authors:** Gebisa Guyasa Kabito, Sintayehu Daba Wami

**Affiliations:** Department of Environmental and Occupational Health and Safety, Institute of Public Health, College of Medicine and Health Sciences, University of Gondar, P.O. Box 196, Gondar, Ethiopia

**Keywords:** Secondary school teachers, Work-related tress, Public schools, Ethiopia

## Abstract

**Objective:**

We conducted a cross-sectional study to assess perceived work-related stress and associated factors among public secondary school teachers in Gondar city, northwest Ethiopia. A self-completed questionnaire was used for data collection. Data was entered into Epi-info version 7.1 and analyzed by SPSS version 20 software. The associations between dependent and independent variables were assessed using a multivariate binary logistic regression analysis based on the adjusted odds ratio (AOR) with 95% confidence intervals (CI) and p values < 0.05.

**Results:**

The response rate was 96.4%. The overall prevalence of perceived work-related stress was 58.2%. Teaching experience < 5 years (AOR 2.03, 95% CI (1.10, 3.73), education level BSC/BED (AOR 1.66, 95% CI (1.07, 3.17), high job demand (AOR 1.61, 95% CI (1.29, 3.74), and poor relationships (AOR 1.88, 95% CI (1.07, 3.31) were significantly associated with work-related stress. In conclusion, the findings showed a high proportion of stress among participants. Therefore, we suggested a need to take action to balance job demand and teaching experience, offering the opportunity to upgrade their educational level and establish good relationships to ease the burden of stress factors related to work.

## Introduction

Stress has been under study since the 1920s [1]. Work-related stress (WRS) is the response that individuals may face when they have job demands and pressures that mismatched their knowledge and abilities [2, 3]. Today, numerous studies have shown teaching is a highly stressful profession worldwide [4, 5]. In European countries, about 10–40% of teachers suffer from extreme stress [6, 7]. Similarly, research in Canada has shown nearly three-fourths of teachers were stressed [8] and with 22% reporting extreme stress in Germany [9]. Moreover, though there is only a small survey in Africa, a high proportion of stress has been identified in Egypt [10], Ethiopia [4], and Nigeria [11].

WRS also leads to issues of occupational health and a major cause of economic loss. For instance, WRS costs US employers $200 billion per year [12]. Indeed, 5274 teachers were absent from Japan’s schools in 2011 due to stress [13]. Moreover, the research in Ethiopia also found that nearly two thirds of teachers planned to leave the profession [14]. Furthermore, this pattern is even more apparent as we move west, Canadian study reported a 40% attrition rate in the first 5 years of teaching due to stress [15].

From a public health perspective, WRS can contribute to physiological disease, decreased well-being and psychological distress [16, 17]. Unless dealt with early, WRS leads to academic decrease and general distress of life including anxiety, depression and suicide [18].

Working experience [19–21], job demands [22], educational level [19, 20], job change [23], lack of support from co-workers, friends and family [23], and poor relationship with colleagues [24] were the most frequently reported factors as major work-related stressors.

Although study into WRS has been a universal move; limited study is currently available in Ethiopia regarding teacher stress. In Ethiopia, the prevalence of stress and factors among teachers in public schools remain very limited. Therefore, this study was aimed to assess perceived work-related stress and associated factors among public secondary school teachers in Gondar city, northwest Ethiopia.

## Main text

### Methods

#### Study design, period and area of study

An institution-based cross-sectional study was employed from March to April 2019. This study was conducted on public secondary school teachers in Gondar city, northwest Ethiopia. Gondar city is located in the northern part of Ethiopia in Amhara National Regional State, at a distance of 747 km from Addis Ababa and 170 km from the Regional capital city Bahirdar. There were a total of 11 public schools and about 711 public secondary schools teachers in Gondar city at the time of data collection.

#### Populations and sample size determination

All public secondary school teachers in Gondar City and working at a selected schools and having worked at least 6 months prior to the study were included as part of the study population, while those on an annual, sick, family or maternity leave were excluded. We used a single population proportion equation [25–27] to calculate the sample size required for the study. With the following assumptions *P* (proportion of stress assumed to be 0.5 since this would provide the maximum sample size), *d* (the permissible Margin of error (the required precision = 5%) and Zα/2 (the value of the standard normal curve score corresponding to the given confidence interval = 1.96) corresponding to 95% confidence level, the minimum sample size (n) was estimated as:

$$n = \frac{{\left( {z\alpha /2} \right)2 p \left( {1 - p} \right)}}{ d2} = \frac{{\left( {1.96} \right)2 \times 0.5 \left( {1 - 0.5} \right)}}{{ \left( {0.05} \right)2}} = 384.16$$

Accordingly, the final sample was 424, considering the 10% non-response rate.

#### Data collection instrument and sampling procedures

A standardized perceived stress scale (PSS-14) [28] questionnaire was used to measure the prevalence of perceived WRS. The scale comprised 14 questions ranging from 0 to 4 each item and ranged from never, almost never, sometimes, fairly often and very often, according to their occurrence respectively, in 1 month before the survey. An example of one of the questions on the measure was “In the last month, how often have you felt nervous and “stressed”?” The PSS-14 has an internal consistency of 0.85 (Cronbach co-efficient) and test-retest reliability over a short retest interval of 0.85 [28]. PSS-14 scores are obtained by reversing scores on four positive items, e.g. 0 = 4, 1 = 3, 2 = 2, etc., and then summing up all 14 items. Items 4, 5, 6, 7 and 10 are positive items. The scale produced a single ranking, with high scores indicated higher stress levels and vice versa. There are also stratified quartiles in the PSS score. The upper two and lower two quartiles were combined (28 being the upper limit operating cut-off value) and were labeled as stressed and not stressed respectively. The cut-off value was chosen in line with a similar study from Egypt [29] and India [30]. This standardized questionnaire has been used in a variety of literature studies, including Ethiopia [31, 32]. UCU (University and College Union) Model Stress Questionnaire was used to test different types of factors of WRS [33, 34]. Specifically, we computed the mean score for all of them (job demand (18 items), (job control (9 items), (job relationships (9 items), (role ambiguity (9 items), (job change (10 items) and (support (9 items). A simple random sampling technique was used to select study participants. Data collectors issued these self-report questionnaires to randomly selected study participants at their office. As part of this study, secondary school teachers in Ethiopia were 9–12 grade teachers.

#### Data quality control

The questionnaire was translated by an expert into Additional file 1: Amharic (local) and returned to Additional file 2: English. One day training was offered for both data collectors and supervisors on topics related to the research objectives, clarity of questions, confidentiality of information and consent in the study. We hired six occupational health professionals working outside the current study area to collect data. Supervisors checked the completeness, quality and consistency of the information collected. In order to test the accuracy and quality of the questionnaire, we conducted a pre-test on 15 samples in a school not included in the final survey. Adjustments were made grounded on the outcomes of the pretest.

#### Data management and statistical analysis

The data were checked for completeness and entered into EPI info version 7 and exported to SPSS version 20 for analysis. Using a binary logistic regression analysis, we fitted each predictor variable in to a bivariate logistic regression model separately to explore associations with the dependent variable (perceived work-related stress). Significant predictors at p-value  < 0.2 in a bivariate analysis were exported to the multivariable logistic regression model using backward variable selection method. Hosmer and Lemeshow goodness-of-fit test was used to check the model fitness (p > 0.05). A multi co-linearity assumption was checked using Variance Inflation Factor (VIF < 4). Lastly, significant association was established at p < 0.05 and adjusted odds ratio (AOR) with 95% confidence intervals (CI) in the multivariable model.

### Results

#### Socio-demographic characteristics of the respondents

In this study, the response rate (409/424) was 96.4%. Of those surveyed, 65.3% were male and 65.8% married. About half, 50.4% of the respondents’ age was 30–39 ranging from 23 to 63 with a mean (± SD) of 36.02 (± 7.33) years. The majority, 74.8% of respondents had a BSc/Bed, whereas about 18.1% had a Master and above educational levels. Nearly a quarter, 26.7% of the respondents had 5 to 10 years of teaching experience, while about 24.4% had > 10 years of teaching experience. Aside this, around one-third of the respondents (29.6%) had a monthly salary of 4501–5500 ETB (Table 1).

**Table 1:** Socio-demographic characteristics of respondents among public secondary school teachers in Gondar city, northwest Ethiopia, 2019 (n = 409)

Variables	Frequency	Percent (%)
Sex		
Male	267	65.3
Female	142	34.7
Age		
≤ 29	83	20.3
30–39	206	50.4
40–49	96	23.5
≥ 50	24	5.9
Educational level		
Diploma	29	7.1
BSc/BED	306	74.8
Master and above	74	18.1
Marital status		
Married	269	65.8
Single	114	27.9
Divorced/separate/widowed	26	6.4
Monthly salary (ETB)		
≤ 4500	107	26.2
4501–5500	121	29.6
≥ 5500	181	24.3
Teaching experience		
< 5 year	99	24.7
5–10	109	26.7
10–15	100	24.4
≥ 16	101	24.7
Religion		
Orthodox	300	73.3
Muslim	65	15.9
Protestant	37	9
Catholic	7	1.7

#### Prevalence and factors related to perceived work-related stress

The overall prevalence of perceived work-related stress in the past 1 months was found to be 58.2% (n = 238) [95%CI (53.8–62.8)].

The multivariable logistic regression analysis showed that less than 5 years of teaching experience, BSc/Bed education level, high job demand, and poor job relations were significantly associated with perceived WRS. Accordingly, respondents with < 5 years of teaching experience were 2.03 times more likely to experience WRS than those with ≥ 16 years of teaching experience [AOR: 2.03; 95% CI (1.10, 3.73)]. The odds of Perceived WRS were 66% times higher among participants with BSc/Bed by their education level [AOR: 1.66; 95% CI (1.07, 3.17)]. The chances of developing WRS were 61% times higher among participants with high job demand compared to their counterparts [AOR: 1.61; 95% CI (1.29, 3.74)]. The probability of experiencing WRS among participants with poor relationships was 1.88 times greater than those with good relationships [AOR: 1.88; 95% CI (1.07, 3.31)] (Table 2).

**Table 2:** Factors associated with perceived work-related stress among public secondary school teachers in Gondar city, northwest Ethiopia, 2019

Variable (n = 409)	Perceived WRS		COR (95%CI)	AOR (95%CI)
	Stressed	Not stressed		
Sex				
Male	154	113	1	1
Female	84	58	1.06 (0.70, 1.61)	1.06 (0.68, 1.66)
Age				
≤ 29	53	30	2.47 (0.98, 6.25)	1.59 (0.44, 5.69)
30–39	116	90	1.80 (0.77, 4.25)	1.91 (0.63, 5.85)
40–49	59	37	2.23 (0.90, 5.54)	2.46 (0.93, 6.52)
≥ 50	10	14	1	1
Educational level				
Diploma	19	10	2.12 (0.87, 5.16)	2.14 (0.84, 5.48)
BSC/BED	184	122	1.68 (1.01, 2.80)	1.66 (1.07,3.17)**
≥ Master	35	39	1	1
Marital status				
Married	147	122	1	1
Single	74	40	1.54 (0.98, 2.42)	1.39 (0.82, 2.35)
Divorced	17	9	1.57 (0.68, 3.64)	1.50 (0.61, 3.68)
Monthly salary (ETB)				
≤ 4500	63	44	1.06 (0.65, 1.72)	0.63 (0.32, 1.27)
4501–5500	71	50	1.05 (0.66, 1.68)	0.93 (0.51, 1.70)
≥ 5500	104	77	1	1
Teaching experience				
< 5 years	71	28	2.04 (1.13, 3.67)	2.03(1.10, 3.73)**
5–10 years	56	53	0.85 (0.49, 1.46)	0.84 (0.48, 1.48)
10–15 years	55	45	0.98 (0.56, 1.71)	0.99 (0.56, 1.77)
≥ 16 years	56	45	1	1
Cigarette smoking				
Smoker	38	23	1.22 (0.70, 2.14)	0.95 (0.48, 1.89)
Not smoker	200	148	1	1
Job demand				
High	135	76	1.64 (1.10, 2.43)	1.61 (1.29,3.74)***
Low	103	95	1	1
Job control				
High	120	118	1	1
Low	82	89	0.91 (0.61, 1.34)	0.90(0.51, 1.56)
Relation ships				
Good	113	125	1	1
Poor	89	82	1.20 (0.81, 1.78)	1.88 (1.07,3.31)*
Role ambiguity				
Yes	114	124	0.81 (0.55, 1.20)	0.62 (0.36, 1.05)
No	91	80	1	1
Job change				
High	123	115	0.90 (0.61, 1.33)	0.66 (0.38, 1.15)
Low	93	78	1	1
Support				
High	141	83	1	1
Low	97	88	0.65 (0.44, 0.96)	0.54(0.32, 1.20)

*AOR* adjusted odds ratio, *CI* confidence interval, *COR* crudes odds ratio

*Statistically significant at p < 0.05, **statistically significant at p < 0.001, ***statistically significant at p < 0.0001, Hosmer and Lemeshow test = 0.920 showed that the model fitted well

### Discussion

The overall prevalence of perceived WRS in this study was found to be 58.2%, which is almost similar to study conducted in Malaysia (55.7%) [35]. However, our finding indicates a higher prevalence compared to the studies in the Malaysia, (32.3%) [36], Libya (39.5%) [37], Ireland (45%) [38], Iran (40.02%) [39],Nigeria (32.9%) [40], and UK (43%) [41]. On the other hand, we found a lower prevalence of work-related stress compared to studies in Hong Kong (91.6%) [42], Nigeria (72.2%) [11], India (69.57%) [43], and Egypt (100%) [44]. Such variations might possibly be due to different local characteristics, including perceptions, traditions, study tools, living standards and educational systems available in these countries, which could have given either exacerbation or buffering effects of stressors related to work [45]. In addition, the disparity could be due to different approaches, periods of research and sample population.

The study result showed that the length of teaching experience was significantly associated with WRS. This outcome was consistent with studies in Macedonia [19] and China [46]. This could be due to the lack of time for newly hired teachers to complete and schedule their classroom tasks and workloads adequately [47]. Moreover, because of their professional role as a teacher, the workload is usually minimized by senior staff due to their ability to assess their role. Furthermore, older teachers may be more seasoned and environmentally adaptable and mature to cope with stress [48]. In addition young teachers in teaching/disruptive students may not be comfortable enough, since they are most likely inexperienced in their career compared to their older staff [43].

The current study showed that the high job demand was statistically significant with WRS. Other studies have supported this result [49–54]. The possible explanation could be that the sheer amount of work that teachers have to do is the one factor that has had a clear impact on stress levels in the profession [50]. Another possible reason could be that the number of students in Ethiopia, including the current area of study, is increasing by an average of 75–82 students per class [55].

Moreover, this study showed that educational level was significantly associated with WRS. Specifically, findings on education level showed that lower educational levels were associated with higher stress; this result is harmonized with studies reported in Kosovo [56], Nepal [57], European countries [58], and Malaysia [36]. One possible reason may be that dealing with certain complexities of the teaching role may be more difficult if there is a lower level of education.

Furthermore, this study showed that poor job relationship was associated with WRS. Results from other studies support our results [24, 54, 59]. A possible reason could be that harmony and positive relationships between teachers may isolate stress triggers [60]. In addition, good relationships could continue to enhance self-esteem and allow teachers not to feel isolated, leading to stressors buffering [61].

### Conclusion

This study showed a high prevalence of perceived WRS. The most important factors found by a multivariable logistic regression model were: teaching experience, job demand, educational level and relationships which were associated with WRS. Therefore, we suggested a need to take action to balance job demand and control, offering the opportunity to upgrade their educational level and establish good relationships to ease the burden of stress factors related to work. We also suggested that other causes of WRS, such as working conditions and further large-scale study, be considered for future research.

## Limitation of the study

The study is limited by its cross-sectional nature, limiting causality inferences, and dependence on self-reporting, resulting in possible over-or under-reporting. Despite these limitations, we feel that the study provides a reasonably accurate assessment of perceived work-related stress and associated risk factors among secondary school teachers.

## Supplementary information

**Additional file 1.** Amharic version of the tool.

**Additional file 2.** English version of the tool.

## Data Availability

The datasets generated and analyzed during this study were included in the main document of this manuscript.

## Abbreviations

AOR: adjusted odds ratio
BSC/BED: Bachelors of Science or Bachelors of Education
CI: confidence interval
COR: crude odds ratio
ETB: Ethiopian Birr
VIF: Variance Inflation Factor
WRS: work related stress

## References

[1] Selye H (1956). The stress of life.

[2] Cavanaugh MA, Boswell WR, Roehling MV, Boudreau JW (2000). An empirical examination of self-reported work stress among US managers. J Appl Psychol.

[3] Health and safety executive: working together to reduce stress at work. 2016. http://www.hse.gov.uk/pubns/indg424.pdf. Accessed 12 Aug 2019.

[4] Gebrekirstos HA (2015). Occupational stress among secondary school teachers and their coping strategies: the case of central zone of tigray region. Int J Acad Res Educ Rev.

[5] Cooper C, Travers C (2012). Teachers under pressure: stress in the teaching profession.

[6] Kristensen TS, Borritz M, Villadsen E, Christensen KB (2005). The copenhagen burnout inventory: a new tool for the assessment of burnout. Work Stress.

[7] Chaplain RP (2008). Stress and psychological distress among trainee secondary teachers in England. Educ Psychol.

[8] Duxbury LE, Higgins C (2012). 2012 National study on balancing work and caregiving in Canada.

[9] Unterbrink T, Hack A, Pfeifer R, Buhl-Grießhaber V, Müller U, Wesche H, Frommhold M, Scheuch K, Seibt R, Wirsching M (2007). Burnout and effort–reward-imbalance in a sample of 949 German teachers. Int Arch Occup Environ Health.

[10] Hassan AM, Rizk SM, El-Naser EMS (2018). Asssessment of work stress and coping strategies among primary school teachers. Med J Cairo Univ.

[11] Asa FT, Lasebikan VO (2016). Mental health of teachers: teachers’ stress, anxiety and depression among secondary schools in Nigeria. Int Neuropsychiatr Dis J.

[12] Hassard J, Teoh K, Cox T, Dewe P, Cosmar M, Gründler R, Flemming D, Cosemans B, Van den Broek K (2014). Calculating the cost of work-related stress and psychosocial risks.

[13] Kataoka M, Ozawa K, Tomotake M, Tanioka T, King B (2014). Occupational stress and its related factors among university teachers in Japan. Health.

[14] Fenot B: Teacher job satisfaction and dissatisfaction: an empirical study of urban teachers in Ethiopia. Doctoral thesis. Teacher College: Columbia University; 2005.

[15] Clandinin DJ, Long J, Schaefer L, Downey CA, Steeves P, Pinnegar E, McKenzie Robblee S, Wnuk S (2015). Early career teacher attrition: intentions of teachers beginning. Teach Educ.

[16] Harmsen R, Helms-Lorenz M, Maulana R, van Veen K, van Veldhoven M (2019). Measuring general and specific stress causes and stress responses among beginning secondary school teachers in the Netherlands. Int J Res Method Educ..

[17] Nieuwenhuijsen K, Bruinvels D, Frings-Dresen M (2010). Psychosocial work environment and stress-related disorders, a systematic review. Occup Med.

[18] Abebe AM, Kebede YG, Mengistu F (2018). Prevalence of stress and associated factors among regular students at Debre Birhan Governmental and Nongovernmental Health Science Colleges North Showa Zone, Amhara Region, Ethiopia 2016. Psychiatry J.

[19] Agai-Demjaha T, Bislimovska JK, Mijakoski D (2015). Level of work related stress among teachers in elementary schools. Open Access Maced J Med Sci.

[20] Alfred Solomon D, David Robinson P, Thephilah Cathrine R (2017). Assess the level of stress among school teachers in selected schools at vellore. Int J Dev Res.

[21] Aftab M, Khatoon T (2012). Demographic differences and occupational stress of secondary school teachers. Eur Sci J.

[22] Hadi AA, Naing NN, Daud A, Nordin R, Sulong MR (2009). Prevalence and factors associated with stress among secondary school teachers in Kota Bharu, Kelantan, Malaysia. Southeast Asian J Trop Med Public Health.

[23] Agai-Demjaha T, Minov J, Stoleski S, Zafirova B (2015). Stress causing factors among teachers in elementary schools and their relationship with demographic and job characteristics. Open Access Maced J Med Sci.

[24] Maphalala MC (2014). The manifestation of occupational stress in the teaching profession: the unheeded voices of teachers. Mediterr J Soc Sci.

[25] Lwanga SK, Lemeshow S, Organization WH (1991). Sample size determination in health studies: a practical manual.

[26] Charan J, Biswas T (2013). How to calculate sample size for different study designs in medical research?. Indian J Psychol Med.

[27] Fosgate GT (2009). Practical sample size calculations for surveillance and diagnostic investigations. J Vet Diagn Invest.

[28] Cohen S, Kamarck T, Mermelstein R (1983). A global measure of perceived stress. J Health Soc Behav.

[29] Amr M, El Gilany AH, El-Hawary A (2008). Does gender predict medical students’ stress in Mansoura, Egypt?. Med Educ Online.

[30] Krutarth RB, Nadeera VP, Prasanna KS, Jayram S (2013). Perceived stress and sources of stress among medical undergraduates in a private medical college in Mangalore, India. Int J Biomed Adv Res.

[31] Madebo W, Yosef T, Tesfaye M (2016). Assessment of perceived stress level and associated factors among health science students at Debre Birehane University, North Shoa Zone of Amhara Region, Ethiopia. Health Care: Curr Rev.

[32] Kasa AS, Tesfaye TD (2017). A study on perceived stress among under graduate medical students of Bahir Dar University, Bahir Bar, North West Ethiopia, 2016: institutional based cross sectional study. J Case Rep Stud.

[33] Kyriacou C (1987). Teacher stress and burnout: an international review. Educ Res.

[34] Kinman G, Wray S (2014). Taking its toll: rising stress levels in further education.

[35] Hanizah M. The effect of information technology usage on the prevalence of stress among school teachers in Selangor and factors affecting the stress. Unpublished Masters Thesis. Universiti Kebangsaan: Malaysia; 2003.

[36] Othman Z, Sivasubramaniam V (2019). Depression, anxiety, and stress among secondary school teachers in Klang, Malaysia. Int Med J.

[37] Taher YA, Samud AM, Hashemi MM, Kabuoli NF (2016). Prevalence of depression, anxiety and stress among libyan primary and secondary school teachers: a cross-sectional study. Jordan J Pharm Sci.

[38] Darmody M, Smyth E (2011). Job satisfaction and occupational stress among primary school teachers and school principals in Ireland.

[39] Ahghar G (2008). The role of school organizational climate in occupational stress among secondary school teachers in Tehran. Int J Occup Med Environ Health.

[40] Okwaraji F, Aguwa E (2015). Burnout, psychological distress and job satisfaction among secondary school teachers in Enugu, South East Nigeria. J Psychiatry.

[41] Phillips S, Sen D, McNamee R (2007). Prevalence and causes of self-reported work-related stress in head teachers. Occup Med.

[42] Chan AH, Chen K, Chong EY. Work stress of teachers from primary and secondary schools in Hong Kong. In: International multi conference of engineers and computer scientists 2010. IMECS 2010; 2010. p. 1903–6. http://dspace.cityu.edu.hk/handle/2031/7097.

[43] Dawn S, Talukdar P, Bhattacharje S, Singh OP (2017). A study on job related stress among school teachers in different schools of West Bengal, India. East J Psychiatry.

[44] Desouky D, Allam H (2017). Occupational stress, anxiety and depression among Egyptian teachers. J Epidemiol Global Health.

[45] Mark GM (2008). The relationship between workplace stress, and Job characteristics, individual differences, and mental health.

[46] Xin W, Talwar P, Wah TK, Yusoff NFM, Bee OY, Ghani KA (2019). Occupational stress among primary school teachers: a study in Jilin province. J Cog Sci Hum Dev.

[47] Anhorn R (2008). The profession that eats its young. Delta Kappa Gamma Bulletin.

[48] Smethem L, Adey K (2005). Some effects of statutory induction on the professional development of newly qualified teachers: a comparative study of pre-and post-induction experiences. J Educ Teach.

[49] Manabete S, John C, Makinde A, Duwa ST (2016). Job stress among school administrators and teachers in Nigerian secondary schools and technical colleges. Int J Educ Learn Devel.

[50] Henshaw P (2017). Teacher stress is most closely linked to high job demands. Seced.

[51] Khan F, Yusoff R, Khan A (2014). Job demands, burnout and resources in teaching a conceptual review. World Appl Sci J.

[52] Mulholland R, McKinlay A, Sproule J (2013). Teacher interrupted: work stress, strain, and teaching role. Sage Open.

[53] Ghulza FH, Qamar ZA, Arshad M, Haider G (2019). A study of the organizational stress among public sector secondary school teachers in Punjab. Eur Online J Nat Soc Sci.

[54] Ravalier J, Walsh J (2018). Working conditions and stress in the english education system. Occup Med.

[55] Bank W (2005). Education in Ethiopia: strengthening the foundation for sustainable progress.

[56] Shkëmbi F, Melonashi E, Fanaj N (2015). Workplace stress among teachers in Kosovo. SAGE Open.

[57] Mondal J, Shrestha S, Bhaila A (2011). School teachers: job stress and job satisfaction, Kaski, Nepal. Int J Occup Saf Health.

[58] Lunau T, Siegrist J, Dragano N, Wahrendorf M (2015). The association between education and work stress: does the policy context matter?. PLoS ONE.

[59] Ekundayo HT, Kolawole AO (2013). Stress among secondary school teachers in Ekiti State, Nigeria. J Educ Soc Res.

[60] Eres F, Atanasoska T (2011). Occupational stress of teachers: a comparative study between Turkey and Macedonia. Int J Humanit Soc Sci.

[61] Reis HT, Clark MS, Holmes JG. Perceived partner responsiveness as an organizing construct in the study of intimacy and closeness. In: Handbook of closeness and intimacy. Psychology Press. 2004: p. 211–36.

