# Supplementary material for: Perceived work-related stress and its associated factors among public secondary school teachers in Gondar city: a cross-sectional study from Ethiopia
Source: BMC Res Notes. 2020 Jan 17;13:36. doi: 10.1186/s13104-020-4901-0 (PMC6969455; doi:10.1186/s13104-020-4901-0)
Supplement: Supplementary file 1 — Additional file 1. Amharic version of the tool. [file 13104_2020_4901_MOESM1_ESM.docx]

በዚህ ልኬት ውስጥ ያሉ ጥያቄዎች በመጨረሻው ዓመት ውስጥ ስለ ስሜቶችዎ እና ሀሳቦችዎ ይጠይቁዎታል። በእያንዳንዱ ሁኔታ ፣ እንዴት እንደሰማዎት ወይም እንዳሰቡት የሚሰማዎትን ምን ያህል የሚወክል ክበብ ላይ “X” በማስቀመጥ ምላሽዎን እንዲያመለክቱ ይጠየቃሉ ፡፡ ምንም እንኳን የተወሰኑት ጥያቄዎች ተመሳሳይ ቢሆኑም በመካከላቸው ልዩነቶች አሉ እና እያንዳንዱን እንደ የተለየ ጥያቄ መያዝ አለብዎት ፡፡ በጣም ጥሩው አቀራረብ በፍጥነት መልስ መስጠት ነው። ማለትም ፣ አንድ የተወሰነ መንገድ የተሰማዎትን ስንት ጊዜ ለመቁጠር አይሞክሩ ፣ ግን ይልቁን ምክንያታዊ ግምት የሚመስል አማራጭን ያመልክቱ።

|  | 1. በጭራሽ | 2.በፍጹም | 3.አንዳንድ ጊዜ | 4.ብዙ ጊዜ | 5.በአብዛኛው ጊዜ |
| --- | --- | --- | --- | --- | --- |
| 1. ባለፈው ወር ባልተጠበቀ ነገር የተነሳ ምን ያህል ጊዜ ተናደ ነበር? |  |  |  |  |  |
| 2.ባለፈው ወር በሕይወትዎ ውስጥ አስፈላጊ የሆኑትን ነገሮች ለመቆጣጠር እንዳልቻሉ ምን ያህል ይሰማዎታል? |  |  |  |  |  |
| 3.ባለፈው ወር ውስጥ ምን ያህል ጊዜ “ውጥረት”ይሰማዎታል? |  |  |  |  |  |
| 4. ባለፈው ወር ውስጥ በየቀኑ የዕለት ተዕለት ችግሮችን እና ብስጭቶችን በተሳካ ሁኔታ እንዴት ተወጥረዋል? |  |  |  |  |  |
| 5.ባለፈው ወር በሕይወትዎ ውስጥ የሚከሰቱትን አስፈላጊ ለውጦችን በተሳካ ሁኔታ እየተቋቋሙ እንደሆኑ የሚሰማዎት ጊዜ ስንት ነበር? |  |  |  |  |  |
| 6.የግል ችግሮችዎን ለማስተናገድ ችሎታዎ ባለፈው ወር ውስጥ ስንት ጊዜ ተማምነው ነበር? |  |  |  |  |  |
| 7.ባለፈው ወር ነገሮች ነገሮች እየሄዱ እንደሆኑ የሚሰማዎት ጊዜ ምን ያህል ነበር? |  |  |  |  |  |
| 8.ባለፈው ወር ውስጥ ማድረግ ያለብዎትን ነገሮች ሁሉ መቋቋም ስለቻሉ ምን ያህል ጊዜ ሆኖ ተገኝተዋል? |  |  |  |  |  |
| 9.ባለፈው ወር በህይወትዎ ውስጥ ብስጭቶችን ለመቆጣጠር ምን ያህል ጊዜ ቆይተዋል? |  |  |  |  |  |
| 10.ባለፈው ወር ውስጥ ፣ ነገሮች በሁሉም ነገር ላይ እንደሆኑ የሚሰማዎት ጊዜ ምን ያህል ነበር? |  |  |  |  |  |
| 11.ከግል ቁጥጥርዎ ውጭ በተከሰቱት ነገሮች የተነሳ ባለፈው ወር ምን ያህል ጊዜ ተቆጥተው ነበር? |  |  |  |  |  |
| 12.ባለፈው ወር ምን ማከናወን ስለሚኖርብዎት ነገር እያሰላሰለዎት ስንት ጊዜ ሆኖዎት ነበር? |  |  |  |  |  |
| 13.ባለፈው ወር ውስጥ ጊዜዎን የሚያጠፉበትን መንገድ ለመቆጣጠር ስንት ጊዜ ተጉዘዋል? |  |  |  |  |  |
| 14.ባለፈው ወር ውስጥ እነሱን ማሸነፍ ለማይችሉ ችግሮች በጣም ከፍተኛ በመሆናቸው ምን ያህል ጊዜ ተሰምተውዎት ነበር? |  |  |  |  |  |
