# Supplementary material for: Perceived work-related stress and its associated factors among public secondary school teachers in Gondar city: a cross-sectional study from Ethiopia
Source: BMC Res Notes. 2020 Jan 17;13:36. doi: 10.1186/s13104-020-4901-0 (PMC6969455; doi:10.1186/s13104-020-4901-0)
Supplement: Supplementary file 2 — Additional file 2. English version of the tool. [file 13104_2020_4901_MOESM2_ESM.doc]

**The questions**

**In this scale ask you about your feelings and thoughts during the last YEAR. In each case, you will be asked to indicate your response by placing an “X” over the circle representing HOW OFTEN you felt or thought a certain way. Although some of the questions are similar, there are differences between them and you should treat each one as a separate question. The best approach is to answer fairly quickly. That is, don’t try to count up the number of times you felt a particular way, but rather indicate the alternative that seems like a reasonable estimate.**

1. **In the last month, how often have you been upset because of something that happened unexpectedly?**
2. **In the last month, how often have you felt that you were unable to control the important things in your life?**
3. **In the last month, how often have you felt nervous and “stressed”?**
4. **In the last month, how often have you dealt successfully with day to day problems and annoyances?**
5. **In the last month, how often have you felt that you were effectively coping with important changes that were occurring in your life?**
6. **In the last month, how often have you felt confident about your ability to handle your personal problems?**
7. **In the last month, how often have you felt that things were going your way?**
8. **In the last month, how often have you found that you could not cope with all the things that you had to do?**
9. **In the last month, how often have you been able to control irritations in your life?**

**Almost Fairly Very**

**Never Never Sometimes Often Often**

1 2 3 4 5

1. **In the last month, how often have you felt that you were on top of things?**
2. **In the last month, how often have you been angered because of things that happened that were outside of your control?**
3. **In the last month, how often have you found yourself thinking about things that you have to accomplish?**
4. **In the last month, how often have you been able to control the way you spend your time?**
5. **In the last month, how often have you felt difficulties were piling up so high that you could not overcome them?**

**Almost Fairly Very**

**Never Never Sometimes Often Often**

1 2 3 4 5
